# Tumour necrosis factor, interleukin-6 and interleukin-10 are possibly involved in *Plasmodium vivax*-associated thrombocytopaenia in southern Pakistani population

**DOI:** 10.1186/1475-2875-13-323

**Published:** 2014-08-16

**Authors:** Afsheen Raza, Muhammad Shahzeb Khan, Najia Karim Ghanchi, Ahmed Raheem, Mohammad A Beg

**Affiliations:** Department of Pathology and Microbiology, Aga Khan University, Stadium Road, PO Box 3500, Karachi, 74800 Pakistan; Dow University of Health Sciences, Civil Hospital, Karachi, 74800 Pakistan

**Keywords:** *Plasmodium vivax*, Thrombocytopaenia, Cytokines

## Abstract

**Background:**

In Pakistan, *Plasmodium vivax* is endemic causing approximately 70% of the malaria cases. A number of haematological changes, especially thrombocytopaenia have been reported for *P. vivax*. Several host factors including cell-mediated immune cells, such as IL-1, IL-6 and IL-10 have been documented for *P. vivax*-induced thrombocytopaenia. However, study on correlation of cytokines and thrombocytopaenia in *P. vivax*, particularly in patients with severe signs and symptoms has not been reported from Pakistan.

**Methods:**

A case control study to correlate TNF, IL-6 and IL-10 in healthy controls and thrombocytopaenic *P. vivax*-infected patients (both uncomplicated and complicated cases) from southern Pakistan was carried out during January 2009 to December 2011. One Hundred and eighty two patients presenting with microscopy-confirmed asexual *P. vivax* mono-infection and 100 healthy controls were enrolled in the study at Aga Khan University Hospital, Karachi. Enzyme-linked immunosorbent assay (ELISA) was performed for determination of TNF, IL-6 and IL-10 levels.

**Results:**

Out of 182 cases, mild thrombocytopaenia (platelet count 100,000-150,000 mm^3^) was observed in ten (5.5%), moderate (50,000-100,000 mm^3^) in 93 (51.1%), and profound thrombocytopaenia (<50,000 mm^3^) was detected in 79 (43.4%) patients. IL-6 and IL-10 levels were found approximately three-fold higher in the mild cases compared to healthy controls. Two-fold increase in TNF and IL-10 (p < 0.0001) was observed in profound thrombocytopaenic when compared with moderate cases, while IL-6 was not found to be significantly elevated.

**Conclusion:**

Cytokines may have a possible role in *P. vivax*-induced thrombocytopaenia in Pakistani population. Findings from this study give first insight from Pakistan on the role of cytokines in *P.vivax*-associated thrombocytopaenia. However, further studies are required to understand the relevance of cytokines in manifestations of thrombocytopaenia in *P. vivax* malaria.

## Background

*Plasmodium vivax* malaria is an important public health problem worldwide causing an estimated 80–215 million clinical cases annually [1]. Malaria is endemic throughout Pakistan with an estimated 4.5 million suspected cases reported by the World Health Organization (WHO). Both *P. vivax* and *Plasmodium falciparum* co-exist, with *P. vivax* being the major contributor (70%) of malaria burden in all areas [1].

Clinical symptoms commonly associated with vivax malaria include fever, malaise, chills, acute respiratory distress (ARDS), acute renal failure, coma, death, while frequent haematological disturbance observed include anaemia and thrombocytopaenia [2]. Various malaria-endemic countries, including Indonesia, Columbia, Kenya, India, and Pakistan, have documented high frequency of thrombocytopaenia in malaria patients [3–13]. Furthermore, case reports describing clinical manifestations of thrombocytopaenia due to *P. vivax* have been described indicating the significance of the respective parameter in vivax malaria [14–21]. Recent studies have also highlighted the usefulness of thrombocytopaenia as a plausible clinical marker of malaria diagnosis with significant sensitivity, specificity, positive, and negative predictive values [22–24].

Thrombocytopaenia in *P. vivax* is frequently observed (24-94%) however; mechanisms associated with malaria thrombocytopaenia are not clear at present. Speculated mechanisms include coagulopathy, splenic sequestration of injured platelets, bone-marrow alterations, platelet aggregation, antibody-mediated platelet destruction and oxidative stress [3, 25]. Association of cell-mediated immune cells, such as interlukin-1 (IL-1), IL-6, IL-10, tumour necrosis factor (TNF) and transforming growth factor-β (TGF-β) and thrombocytopaenia have been documented in various studies [3, 26–28]. There is a paucity of baseline data on various aspects of *P. vivax*, including cytokine profile, correlation of immunological response and haematological manifestations. Therefore, the aim of this study was to determine and correlate baseline levels of TNF, IL-6 and IL-10 in healthy controls and thrombocytopaenic *P. vivax*-infected patients from southern Pakistan. The cytokines data presented in this study is part of ongoing project previously published baseline profiles of biomarkers in malaria patients. (37) In this study cytokine data was used for correlation with thrombocytopaenia.

## Methods

### Study design, settings and case definitions

A case control study using plasma samples from well characterized groups suffering from *P. vivax* infection including uncomplicated cases (n = 100), complicated cases (n = 82) and healthy controls (n = 100) was conducted during January 2009 to December 2011 at The Aga Khan University and Hospital, Karachi. Baseline plasma levels of TNF, IL-6 and IL-10 were compared and correlated between healthy controls and thrombocytopaenic *P. vivax*-infected patients (both complicated and uncomplicated cases). Thrombocytopaenia was categorized as mild, moderate and profound on the basis of reference ranges used in The Aga Khan University and Hospital, Karachi.

Reference ranges for thrombocytopaenia:

- Mild: platelet count range between 100,000 mm^3^ and 150,000 mm^3^
- Moderate: platelet count range between 50,000 mm^3^ and 100,000 mm^3^
- Profound: platelet count range <50,000 mm^3^

### Case definitions

#### Healthy controls

Individuals tested negative on screening test for Hepatitis B, C, human immunodeficiency virus (HIV), syphilis, malaria and having platelet counts >150,000 mm^3^ were recruited as healthy controls.

#### Uncomplicated malaria

Febrile patients tested slide and PCR positive for *P. vivax* infection but no malarial complication were recruited as uncomplicated cases.

#### Complicated malaria

Patients tested slide and PCR positive for *P. vivax* infection and admitted to the Aga Khan University Hospital, Karachi with one or more symptoms of complicated malaria (WHO guidelines) were enrolled as complicated cases.

Both complicated and uncomplicated cases were further stratified on the basis of thrombocytopaenia status. Cases and controls with no co-morbid/associated diagnosis were enrolled in the study. Pregnant women were excluded from the study.

### Ethical considerations

The study was approved by the Ethical Review Committee of Aga Khan University Hospital and conducted in accordance with the Good Clinical Practice of Declaration of Helsinki [29]. Informed consent was obtained from enrolled patients.

### Sample collection and microcopy

Approximately 2 ml of intravenous blood sample in EDTA tube was collected. Initial presence of malaria parasites was established by Leishman’s staining while further species identification was determined by Giemsa staining of thick and thin blood smears [30]. Plasma was collected by centrifuging remaining blood at 3,500 rpm for 15 minutes. Blood and plasma aliquots were stored at -80°C until further analysis. Platelet count of cases and controls was performed using coulter counter (Beckman Coulter Inc, USA).

### DNA extraction and PCR

DNA was extracted from 200 μl of whole blood using QiAamp DNA Mini Kit according to manufacturer’s instructions (Qiagen, USA). Confirmation of *P. vivax* mono-infection was performed using a species-specific PCR [31].

### ELISA for quantification of inflammatory cytokines

TNF, IL-6 and IL-10 were detected in plasma of healthy controls and cases by using standards and ELISA reagents obtained from Endogen (Rockford, IL, USA). Cytokines were measured using a sandwich ELISA technique according to the manufacturer’s instructions and as reported previously [32]. Recombinant human cytokine was used to obtain a dose response curve with a range of detection from 3.9-1,000 pg/ml. All experimental samples were tested in duplicate.

### Statistical analysis

Data were entered in Microsoft Excel and Graph Pad Prism version 5.0 was used for performing further analysis. Arithmetical means and medians were calculated, where applicable, for all continuous baseline demographic variables. Kruskal-Wallis test with Dunn’s multiple comparison was used to compare concentrations of cytokines between study groups. Mann Whitney *U* test was used to verify differences between cases and controls. Correlation between cytokines and thrombocytopaenia in complicated cases was performed using Spearman’s Rank correlation analysis.

## Results

### Baseline demographics

A total of 200 microscopically confirmed *P. vivax* cases were enrolled in the study. Amongst these, 182 samples tested PCR positive for *P. vivax* mono-infection and were thus further analysed. One-hundred healthy controls were also enrolled for the study.

### Thrombocytopaenia profile

Thrombocytopaenia of varying intensity was detected in all cases. Out of 182 cases, mild thrombocytopaenia was observed in ten (5.5%), moderate thrombocytopaenia in 93 (51.1%), and profound thrombocytopaenia was detected in 79 (43.4%) patients. Comparison of haematological parameters between moderate and profound thrombocytopaenia groups showed significant decrease in haemoglobin and red blood cell count while other parameters did not show any significant difference between the study groups. Baseline demographics and haematological parameters of healthy controls and enrolled patients are given in Table 1.

**Table 1:** **Baseline demographics and hematological parameters of the study groups**

	Healthy Controls (HC)	Malaria cases (thrombocytopaenia)			P value
		Mild (MI)	Moderate (M)	Profound (P)	M ***vs***P
**N**	100	10	93	79	
**Haemoglobin**	12.29 ± 1.99	12.38 ± 1.80	12.65 ± 1.63	11.68 ± 2.19	0.002*
**RBC**	4.38 ± 0.43	4.46 ± 0.69	4.39 ± 0.50	4.05 ± 0.70	0.000*
**WBC**	5.94 ± 2.43	7.05 ± 2.31	5.99 ± 2.72	5.8 ± 3.11	0.658
**Neutrophils**	63.5 ± 15	59.38 ± 12.3	72.0 ± 13.78	68.44 ± 14.2	0.098
**Lymphocytes**	21.7 ± 11.4	26.56 ± 9.93	19.25 ± 11.16	22.27 ± 11.19	0.079
**Eosinophils**	0.97 ± 1.16	0.66 ± 0.55	0.82 ± 1.22	0.92 ± 0.96	0.554
**Monocytes**	8.98 ± 5.07	12.94 ± 3.24	7.62 ± 4.6	8.28 ± 5.42	0.387
**Platelets**	175 ± 45.78	102.60 ± 32.97	74.76 ± 27.1	32.17 ± 11.19	0.000*

Values represent medians ± standard deviation.

Reference units used:

Haemoglobin = gm/dl; RBC = 10^12^/L; WBC = 10^6^/L; Platelets = 10^9^/L.

Neutrophils, lymphocytes, eosinophils, monocytes =%.

* = singificant (p-value <0.05).

### Baseline cytokine levels between healthy controls, uncomplicated and complicated *Plasmodium vivax*thrombocytopaenic cases

Median concentrations of TNF, IL-6 and IL-10 were compared between healthy controls and thrombocytopaenic uncomplicated and complicated *P. vivax* cases. Comparison of TNF level between healthy controls and and uncomplicated mild cases did not show any significant difference in mild thrombocytopaenic group, while it was found to be elevated approximately two-fold in moderate and profound group. In complicated cases, TNF was found to be approximately three fold elevated in profound group. IL-6 was found to be 7 fold increased while IL-10 levels were found to be > 20 fold higher in the respective groups. Comparison of profound thrombocytopaenia between uncomplicated and complicated cases showed a two-fold increase in TNF and IL-10 (p < 0.0001) while IL-6 was not found to be significantly elevated (Table 2).

**Table 2:** **Comparison of cytokine levels between healthy controls, uncomplicated and complicated** ***Plasmodium vivax*** **cases**

Cytokines	Healthy controls	Uncomplicated malaria			Complicated malaria			P-value
		Mild	Moderate	Profound	Mild	Moderate	Profound	UM vs CM
**TNF**	178 (57–296)	165 (116–310)	235 (168–647)	482 (320–685)	177 (110–306)	249 (181–664)	652 (339–705)	<0.0001*
**IL-6**	87 (79–93)	215 (219–357)	229 (105–1,107)	313 (149–858)	238 (225–428)	253 (123–1,118)	438 (157–855)	0.352
**IL-10**	37 (21–88)	498 (316–825)	1,079 (428–2,152)	2,552 (995–6,400)	556 (326–886)	1,223 (459–2,269)	4,250 (958–6,750)	<0.0001*

Values represent medians with interquartile ranges (25th and 75th percentile). Mann–Whitney *U* test used to determine significant differences between groups. * = singificant (p-value <0.05).

### Correlation of cytokines and thrombocytopenia in complicated *Plasmodium vivax*cases

Spearman Rank correlation analysis was performed in complicated *P. vivax* cases exhibiting mild, moderate and profound thrombocytopaenia to determine whether complex interactions between cytokines and thrombocytopaenia exist in complicated *P. vivax* cases. No significant correlation was observed between cytokines levels and mild thrombocytopaenia. However, significant negative correlation was observed between IL-6 and IL-10 (p = 0.02) in moderate cases. In profound cases, highly significant positive correlation was observed between TNF and IL-10 (p < 0.0001) while highly significant negative correlation was observed between IL-6 and IL-10 (p < 0.0001) (Table 3).

**Table 3:** **Correlation of cytokines with thrombocytopaenia in complicated** ***Plasmodium vivax*** **cases**

	IL-6	IL-10	TNF-α
**Mild**			
IL-6	1.00	0.756	-0.108
IL-10		1.00	0.764
TNF- α			1.00
**Moderate**			
IL-6	1.00	-0.02*	-0.158
IL-10		1.00	0.158
TNF			1.00
**Profound**			
IL-6	1.00	-0.0001**	0.0001**
IL-10		1.00	0.336**
TNF			1.00

Spearman rank correlation was performed. p <0.05 considered significant.

* = significant.

** = highly significant.

### Comparison of severe symptoms with thrombocytopaenia

Comparison of severe signs and symptoms in complicated cases with thrombocytopaenia revealed that profound thrombocytopaenia was most common in patients suffering from respiratory distress/pulmonary oedema and metabolic acidosis (Table 4) while moderate thrombocytopaenia was observed in patients suffering from jaundice and haemoglobinuria.

**Table 4:** **% positivity of thrombocytopaenia in complicated cases**

Complications	No. of patients	% positivity
Pulmonary oedema/respiratory distress	31	11.2
Metabolic acidosis	20	6.8
Jaundice	26	21.8%
Haemoglobinuria	5	5.2%

## Discussion

In this study, baseline levels of cytokines were examined in healthy controls, uncomplicated and complicated *P. vivax* cases exhibiting varying intensities of thrombocytopaenia. Furthermore, cytokines levels were correlated with thrombocytopaenia status in complicated cases to determine whether complex interactions exist between these molecules and manifestations of disease symptoms in thrombocytopaenic *P. vivax* cases. In this study, thrombocytopaenia was detected in all cases. Moderate and profound thrombocytopaenia was most frequently observed (51.1 and 43.4%, respectively) while mild thrombocytopaenia was observed in only 5.5% of the patients. This finding is consistent with previously reported observations from South America and south Asia where *P. vivax* is endemic [2]. Clinical complications such as disseminated intravascular coagulation (DIC), platelet associated IgG increase (PAIgG), immune thrombocytopaenia purpura (ITP), acute renal failure, pulmonary oedema, splenomegaly, cerebral malaria, and seizures have been reported in *P. vivax* cases exhibiting profound thrombocytopaenia [15–19, 21]. In these cases, bleeding was observed in a few cases, indicating that thrombocytopaenia in *P. vivax* does not always lead to bleeding complications. In this study, complications including metabolic acidosis, pulmonary oedema, jaundice, haemglobunuria were observed in 45% (82/182) of the patients. Of these, profound thrombocytopenia was observed in 18% of the patients of which 11.2% suffered from respiratory distress/pulmonary oedema, while 6.8% suffered from metabolic acidosis. Moderate thrombocytopenia was observed in the remaining cases. Bleeding was observed infrequently in complicated cases exhibiting profound thrombocytopenia indicating that *P. vivax*-associated thrombocytopaenia in Pakistan demonstrates a similar trend of low bleeding tendencies as observed worldwide.

To determine the role of cytokines in thrombocytopaenia, baseline concentrations of pro- and anti-inflammatory cytokines TNF, IL-6, IL-10 were evaluated in healthy controls, uncomplicated and complicated *P. vivax* cases exhibiting mild, moderate and profound thrombocytopaenia. A significant increase of TNF level was observed between uncomplicated and complicated cases exhibiting profound thrombocytopenia while no significant difference was observed between healthy controls and mild cases. This finding corroborates with previously reported data in which thrombocytopaenia was observed in patients exhibiting high TNF levels [33]. It has been postulated that TNF induced thrombocytopaenia is a result of platelet trapping and consumption that occurs in inflamed blood vessels [34]. In *P. falciparum*, this process is well documented and has been associated with immunologically mediated endothelial activation that allows platelet adherence via endothelial adhesion molecules on the blood lining [35]. In *P. vivax*, thrombocytopaenia due to platelet consumption via endothelial adherence/destruction and coagulopathy has also been documented [36, 37]. Elevated levels of TNF in this study imply a similar role of this cytokine in *P. vivax*-associated thrombocytopaenia. However, further studies are required to understand the role of TNF in platelet consumption.

Insignificant increase in IL-6 levels (p value = 0.352) was observed in uncomplicated and complicated cases exhibiting profound thrombocytopaenia. This finding concurs with previous studies that document the role of IL-6 in platelet production [38, 39]. Increased IL-6 levels have been reported in patients with reactive thrombocytosis [40–42]. Furthermore, administration of IL-6 in humans has been associated with increase in circulating platelet counts [43–49]. Thus, a finding of non-significant increase in IL-6 levels in mild, moderate and profound thrombocytopaenic cases implies that regulation of platelet production by IL-6 is disturbed in *P. vivax* cases leading to worsening thrombocytopaenia status.

A significant increase of IL-10 in moderate and profound uncomplicated and complicated *P. vivax* cases was observed in this study. This finding corroborates with previously reported data that reports elevated IL-10 levels in malaria thrombocytopaenia [3, 25, 33]. Furthermore, decreased platelet production in humans in response to administration of recombinant IL-10 has also been documented. The same study postulated that IL-10-induced reduction in platelet count is possibly due to reduction in platelet production [50]. It is possible that similar mechanism of reduced platelet production due to high IL-10 levels is responsible for *P. vivax*-associated thrombocytopaenia in this study. Regulatory cytokines, such as IL-10, are required to reduce the risk of severe disease or more tissue injury in malaria. Therefore, intrinsic IL-10 response induced in response to protecting the host against injury results in down regulation of platelet production mechanisms, thus leading to manifestation of thrombocytopaenia.

To understand complex interaction between cytokines and platelet counts in complicated cases, Spearman rank correlation analysis was performed. It was observed that negative correlation existed between IL-6 and IL-10 in both moderate and profound cases indicating that as IL-10 levels increase, IL-6 levels decrease leading to reduction/disturbance in platelet production. Consequently, this elevated anti-inflammatory bias results in aggravation of thrombocytopaenia in *P. vivax* cases. A significant positive correlation between IL-10 and TNF-α in profound cases further strengthens the role of respective cytokines in platelet consumption, trapping and dysregulation of platelet production. Thus, findings from this study corroborate previously reported data and indicate a possible role of pro- and anti-inflammatory cytokines in manifestation of thrombocytopaenia in *P. vivax* malaria.

Though this study was performed and analyzed keeping all aspects in perspective, however, there are certain limitations in this study. Reference ranges for thrombocytopenia utilized for this study were derived from those used in The Aga Khan University laboratory. Secondly, parasitaemias and patient data, such as days of fever and temperature at admission, was not documented and, therefore, could not be used for analysis as done previously in other studies [5, 51, 52]. Hence, it was not possible to evaluate differences in parasitaemia in relation to thrombocytopaenia and cytokine levels. Furthermore, some studies have also reported age to significantly affect platelet count in malaria patients. In this study, the average age of the patients was between 33–42 years. Since no significant differences with respect to age were observed therefore data was not analyzed for age. However, to better understand the dynamics of thrombocytopenia and cytokine levels future studies focusing on all these aspects in *P. vivax* is suggested.

## Conclusion

Findings from this study give an insight from Pakistan on the role of cytokines in *P. vivax*-associated thrombocytopaenia. However, further studies are required to understand the relevance of cytokines in manifestations of thrombocytopaenia in *P. vivax* malaria.
